# Biosensors in dental, oral and craniofacial applications

**DOI:** 10.1038/s44328-026-00079-w

**Published:** 2026-02-19

**Authors:** Yongchen Tai, Yunshen Li, Kayla M. Mornay, Madison K. Woodard, Wenting Wang, Xianrui Yang, Jing Pan

**Affiliations:** 1 Mechanical and Aerospace Engineering, Herbert Wertheim College of Engineering, University of Florida, Gainesville, FL USA; 2 Department of Orthodontics, College of Dentistry, University of Florida, Gainesville, FL USA

**Keywords:** Biochemistry, Biological techniques, Biomarkers, Biotechnology, Health care

## Abstract

Oral and dental health is an important indicator and determinant of an individual’s overall well-being. Untreated oral diseases can lead to severe systemic complications. Monitoring the oral environment and identifying biochemical and physiological patterns associated with disease states, such as periodontitis, gingivitis, caries, and oral cancers, is essential for early diagnosis and effective intervention. This review evaluates the current clinical needs in biochemical and physiological monitoring for oral healthcare and state-of-the-art biosensors capable of continuous analyte measurement. We surveyed the relevant biomarkers for common oral and dental diseases in patients compared to healthy controls. The design and performance of recent biosensing devices for these target analytes are reviewed and evaluated. For biochemical sensing, we found intraoral biosensors for high-abundance small molecules, such as ions and metabolites, have advanced significantly in recent years. However, robust sensing technologies for low-abundance analytes, including cytokines and other inflammatory biomarkers, remain limited and require further development in sensing mechanisms, bio-interfaces, and device integration. For physiological sensing, particularly the measurement of forces in tooth movement, recent developments in force sensor technologies have substantially improved measurement accuracy over traditional techniques. Despite these advancements, current platforms still face limitations in achieving long-term, real-time monitoring of mechanical conditions within the oral cavity due to challenges related to biocompatibility and device miniaturization. In conclusion, while notable progress has been made in biosensing for oral applications, continued research in device integration with clinical practices is essential to realize robust and clinically deployable biosensor systems that can advance precision oral healthcare.

## Introduction

Oral and dental health is reflective of an individual’s overall health and well-being. When compromised, it can lead to significant systemic complications. In 2025, approximately 3.7 billion people suffered from oral diseases, accounting for nearly half of the global population^1^. Deteriorating dental and oral health can significantly reduce quality of life and impact confidence^2^. Diseases of the stomatognathic system include the jaw, teeth, and soft tissues of the oral cavity. These diseases that affect the global population can include periodontal disease, craniofacial anomalies, head and neck cancers, craniofacial benign tumors, caries, and malocclusion^2^. Monitoring the progression of pathological processes in the mouth is vital for maintaining a stable oral microbiome to prevent the establishment and progression of disease. Despite ongoing efforts to reduce the high prevalence of dental diseases, the oral healthcare system continues to fall short in meeting population needs, limiting access to appropriate dental services, and a population burdened with untreated dental diseases^3^.

Accurate diagnosis is essential to prevent irreversible oral damage, yet current clinical practice relies on symptomatic patient visits and sporadic^2^, lab-based diagnostics that are costly, slow, and offer only intermittent snapshots of biomarker levels and disease states. These intermittent measurements generate sparse clinical data that fail to guide timely intervention^4^ or continuous monitoring. Socioeconomic, systemic, logistical, and geographic barriers^5^ further delay care, leading to advanced disease stages, patient noncompliance, and suboptimal outcomes. The combined challenges highlight the need for low-cost, user-friendly technologies capable of noninvasive^6^, continuous biomarker monitoring and personalized oral health management.

Biosensing technology offers a transformative opportunity to overcome current limitations in the clinical management of oral and dental health. Recent advances in portable and wearable biosensors^7^ have facilitated the integration of sensing elements into the oral environments via intraoral patches^8^, smart toothbrushes^9^, mouthguards^10–13^, dental implants^14,15^, and salivary diagnostic devices^16,17^. While several other notable wearable platforms, such as smart glasses, watches, and contact lenses^18^ have demonstrated utility in health monitoring, the oral cavity presents a uniquely accessible site rich in biofluids, particularly saliva, which contains biomarkers reflective of both local and systemic physiology. The global demand for biosensing technologies has a projected market value of up to $70 billion USD^7,19^, which underscores their potential in facilitating the shift from population-centered to patient-centered care. In clinical practice, these devices could enable real-time detection of disease-associated biomarkers, such as inflammatory cytokines, glucose, pH, and bacterial metabolites, allowing early identification of conditions like periodontitis, caries, and systemic metabolic disorders. By capturing patient-specific molecular signatures, biosensing technologies enhance diagnostic accuracy and support timely, targeted interventions, ultimately improving patient outcomes and preventing disease progression.

Despite advances in oral healthcare and sensing technology development, there remains a critical knowledge gap in understanding how biosensing technologies can be effectively applied to address unmet diagnostic and monitoring needs in oral health applications. A systematic review is therefore needed to identify opportunities, challenges, and translational pathways for sensor integration across various oral health applications. This review first examines the clinical needs and technical challenges associated with the application of biosensors in dental, oral, and Craniofacial applications. It then focuses on the use of biosensing technologies for detecting disease-specific biomarkers, with emphasis on their roles in managing periodontal disease, monitoring orthodontic treatment, and supporting early diagnosis of oral cancer and other dental diseases. Additionally, this paper surveys current biosensor design strategies, highlighting key limitations in sensitivity, biocompatibility, and integration within the oral environment. Finally, it explores emerging directions in dental biosensing, outlining opportunities for innovation that can enhance diagnostic precision and therapeutic outcomes.

## Methodology

A structured literature search was conducted to identify relevant publications that discussed biosensors in the dental, oral, and craniofacial environments as well as various clinical conditions, such as periodontitis, gingivitis, peri-implantitis, oral cancer, dental fluorosis, caries, edentulism, craniofacial anomalies, malocclusion, and temporo-mandibular disorder (TMD). Electronic databases, including Google Scholar and PubMed, were used to locate relevant systematic reviews, randomized controlled trials, review papers, and case studies.

Keywords such as, “periodontitis”, “periodontal disease”, “gingivitis”, “peri-implantitis”, “oral cancer”, “squamous cell carcinoma”, “fluorosis”, “caries”, “cavities”, “craniofacial anomalies”, “malocclusion”, “temporomandibular disorder”, “TMD”, “dental biosensor”, “biomarkers”, “dental biomarkers”, “biosensors” and “Wearable biosensor” were used individually and in combination for an initial broad search. There was no publication year restriction.

Articles were manually evaluated based on relevance and accessibility of the full text. Priority was given to articles that were published within the past 10 years to ensure accurate data and technologies; however, older studies were also considered when appropriate.

The following inclusion criteria were considered for eligible studies for this review paper: (1) discussion of biosensor technology applied to dental, oral, or craniofacial health; (2) studies available in full text; (3) publications in full English text; and (4) provision of qualitative or quantitative data surrounding biosensors or relevant oral conditions.

Due to this paper being a review, no risk of bias tool was applied; however, data collection and research were conducted in systematically to ensure a thorough and comprehensive scope of knowledge.

## Clinical needs and technical challenges

**Fig. 1:**
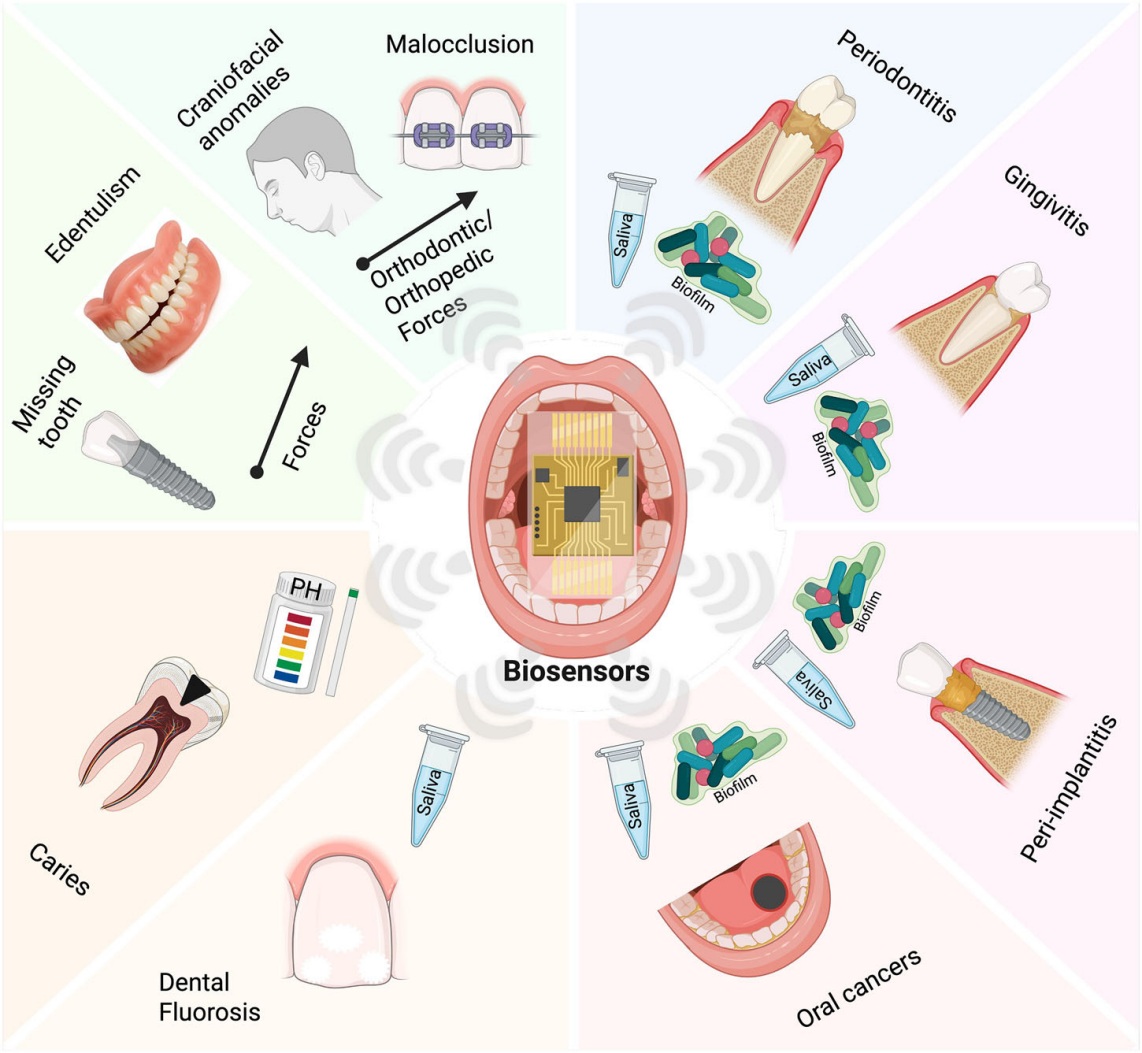
Overview of biosensors in dental, oral, and craniofacial clinical applications. Biosensors can detect biochemical markers of diseases through biofluids, such as saliva and microbial biofilm. They can also detect changes in pH values and mechanical forces. Clinicians can leverage the sensing data to support the early diagnosis and personalized management of various disease states in the oral cavity, such as excessive inflammation, dental caries, implants, and dentures for missing tooth and edentulism, malocclusions, and craniofacial anomalies.

The oral cavity serves as a unique interface between the human body and the external environment, providing direct access to the respiratory and digestive systems, while maintaining close anatomical and functional connections to the circulatory and nervous systems. In addition to its functional and physiological significance, the oral cavity harbors its own microbiome, creating a diverse ecosystem of bacteria, viruses, microeukaryotes, and archaea, all of which play significant roles in oral and overall health^20^. Disruptions and diseases in this environment are increasingly linked to a heightened risk of systemic conditions, such as cardiovascular disease^21^. Many diseases in the oral cavity remain undetected until they have progressed to an advanced stage, which often creates severe, even irreversible damage. Microorganisms are the root cause of diseases such as dental caries, periodontal disease, and gingivitis^22^. Early detection and identification of the infection burden, as well as the continuous tracking of biomolecular changes in the oral environment, such as pH or inflammation, are key for optimal patient prognosis and treatment. For example, studies have reported that simple oral visual screening of the head, neck, and oral cavity to detect the presence of suspicious lesions can reduce mortality in individuals with significant risk factors^23^. For more thorough inspections, histopathology and diagnostic imaging are commonly adopted in clinical practice but come at a great economic burden to patients^23^. Additionally, understanding the body’s response to forces or prosthetic interventions, such as the 3D spatiotemporal forces and subsequent mechanotransduction and inflammatory responses, is important for personalized treatment of malocclusions and management of implants and dentures for teeth replacement. These clinical needs highlight the importance of developing advanced biosensing technologies that are efficient, cost-effective, and targeting a diverse range of analytes (Fig. 1). In this section, we review clinically important disease biomarkers and the technical challenges associated with the detection and continuous tracking of these biomarkers.

### Infection and inflammation

Microorganisms adhere to the tooth structure and can accumulate into biofilms, which cause dental caries and lead to inflammatory diseases such as gingivitis and periodontitis^24^. Detecting biochemical markers in the oral environment can help quantify and monitor the infection burden and host response^25^. Levels of biochemicals, such as cytokines, enzymes, and metabolites, have shown clinical utility in the diagnosis and treatment of various oral diseases^26^. The biomarkers for dental and oral disease are listed in Table 1.

**Table 1:** Biomarkers for dental and oral diseases

Disease	Biomarkers	Ref
Periodontitis	IL-6, IL-17, IL-8, TNF-a, MMP-9, MMP-8, ALP, OPG, CRP	^27,96,102,111–113,117,118,218^
Gingivitis	IL-6, IL-1β, CRP, MMP-8, CRP	^104,112,118,219^
OSCC	IL-8, IL-6, MMP-12	^97,103,115^
OIIRR	IL7, IL-10, IFN- γ	^54^
Caries	*Streptococcus mutans*, glucose, lactic acid, sAA, ammonia, urea, calcium, phosphate, fluoride, pH	^25,42,43,119,131,136,138,150–152,160,161,166^

#### Periodontitis

Periodontitis affects nearly 42% of the United States, making it one of the most prevalent chronic inflammatory diseases^27^. The systemic manifestations of periodontitis are associated with a myriad of diseases, such as Alzheimer’s, chronic kidney disease, diabetes, respiratory tract infections, obesity, and even adverse pregnancy outcomes^28,29^. Clinical signs of periodontitis involve persistent bacterial-induced inflammation, degradation of the periodontal ligament, alveolar bone loss, and eventually tooth loss^30^. A significant contributor to disease progression is the elevated presence of the Gram-negative red complex bacteria *Porphyromonas gingivalis*, *Treponema denticola*, and *Tannerella forsythia*, which are associated with tissue destruction^31^. Periodontal disease can progress with only mild gum bleeding, making early detection by traditional clinical examination unreliable^32^. Monitoring the bacterial load and enzymatic markers of disease pathogenesis is needed for effective diagnosis and treatment. Enzymes such as matrix metalloproteinases-8 (MMP-8) and MMP-9, which mediate collagen breakdown, are correlated with disease severity. Studies have shown the levels of MMPs combined with pro-inflammatory cytokines, such as interleukin-1β (IL-1β), can improve the sensitivity in predicting periodontitis by 74%^27^. Additional biomarkers, including cytokines (e.g., IL-6, IL-8, and TNF-α), enzymes, and proteins involved in bone remodeling (e.g., cathepsin and receptor activator of nuclear factor kappa-B ligand or RANK ligand) are also important biomarkers for clinical assessment and disease management.

#### Gingivitis

Gingivitis represents an early and potentially reversible stage of periodontitis^33,34^. It is a disease that involves gingival inflammation due to the accumulation of microbial communities, often resulting from inadequate oral hygiene practices^35,36^. The presence of infection leads to the recruitment of immune effector cells and the release of pro-inflammatory mediators. Enzymes such as MMPs, alkaline phosphatase (ALP), and acute-phase proteins like C-reactive protein (CRP) are also elevated during gingival inflammation. As gingivitis is a precursor of periodontitis, many of the biomarkers used for periodontitis are also relevant, detectable, and clinically needed in the management of gingivitis.

#### Peri-implantitis

Dental implants are susceptible to inflammatory conditions that closely resemble diseases affecting natural teeth^37^. Peri-implantitis has a similar pathogenesis as periodontitis, resulting in the progressive inflammation and degradation of the supporting bone, significantly reducing implant stability^38^. Due to similar pathological mechanisms, periodontitis and peri-implantitis share many of the same biomarkers. Although clinical diagnosis of peri-implantitis is common, the current diagnostic approach suffers from limited precision and delayed detection, which increases the risk of implant failure^39^.

#### Caries

Dental caries is one of the most widespread diseases globally^40,41^. It develops when *Streptococcus mutans* metabolizes dietary sucrose, producing lactic acid that lowers pH in the local environment, leading to enamel demineralization and cavity formation^25^. There is a wide variety of biomarkers identified to indicate the presence of caries activity, such as salivary α-amylase (sAA)^25^. sAA has been shown to increase in the presence of dental caries and could serve as a diagnostic biomarker for assessing caries risk^42^. Other biomarkers that can be used for the detection of dental caries include ammonia, urea, pH, and ions like calcium, phosphate, and fluoride. Low pH is a strong indicator of active caries as it results directly from acidogenic bacterial metabolism. Conversely, elevated levels of ammonia or urea are indicative of patients with low-caries activity, as these are protective against the development of caries^43^.

### Oral cancer

Oral cancer, such as squamous cell carcinoma (OSCC), ranks among the top six most prevalent cancers worldwide and has seen an 11% increase in mortality between 1990 and 2021^44,45^. The early symptoms of oral cancer are mild or even absent, so patients may not seek care until the disease is advanced, leading to poor prognosis and survival^46^. Given its prevalence and aggressive nature, there is a critical need to understand and monitor biomolecular patterns associated with oral cancer that have high specificity and sensitivity. Several biomarkers have shown strong diagnostic potential for OSCC^47^. IL-8 is among the most studied, with a well-established role in angiogenesis and tumor metastasis^47^. Elevated levels of IL-8 and IL-1β have demonstrated the ability to distinguish not only between healthy and malignant tissue but also between advanced cancer stages (III vs. IV)^48^. Similarly, IL-6, which is associated with tumor-promoting signaling pathways, is often elevated in both serum and saliva of OSCC patients^49^. Additional biomarkers investigated before include MMP-9, which contributes to extracellular matrix degradation and tumor invasion, and lactate dehydrogenase, a metabolic enzyme linked to cell proliferation and membrane disruption in cancerous tissues^47^. Most available studies rely on single-point or sparse measurements of these disease markers. Because oral cancer progression is a dynamic process, capturing the time-resolved fluctuation of biomarker levels is also clinically needed for early detection, staging, and personalized treatment of oral cancer.

### Fluorosis

Fluoride is a nutrient beneficial for healthy growth, but excessive intake over extended periods of time can lead to altered oral microbiome, weakened bone and teeth, and chronic dental and skeletal fluorosis^25,50^. Recognition of different types of fluorides and compounds, such as CaF_2_, KF, and SrF_2_ in water and the oral environment can help monitor dental fluorosis^51^. Detecting increased presence of ions such as F⁻, Ca²⁺, and PO₄³⁻ could indicate fluorosis is present in the oral environment and help monitor treatment progress.

### Malocclusion and craniofacial anomalies

Malocclusion and craniofacial anomalies are managed through orthodontic and orthopedic treatments that rely on controlled force application to guide tooth movement and improve function and esthetics^52^. Management and monitoring of forces in orthodontics and dentofacial orthopedics is imperative in correcting misalignment of the teeth and jaw. Both the force applied during treatment and patient compliance to treatment protocols are important to achieve optimal outcomes^53^. For example, bond failure between the orthodontic bracket and the tooth structure may be neglected by patients, which leads to deviation of applied forces from the treatment plan. However, accurate measurement of the 3D loading forces on teeth and longitudinal monitoring of force adaptation during orthodontic movement is technically challenging and not available in clinical practices today. Prolonged excessive forces in orthodontic tooth movement can cause root resorption, tooth mobility, and damage to the alveolar bone, while inadequate force reduces treatment efficacy. For example, in patients with orthodontically induced inflammatory root resorption (OIIRR), they displayed an increase of pro-inflammatory salivary cytokines such as IL-7, and IFN-γ^54^. Although these cytokines are present during force movements in patients with OIIRR, they can also be induced by other factors, suggesting a need for further research assessing the relationship with these cytokines^54^. Although multiple pursuits have been made in determining orthodontic forces via animal experiments, in vitro sensor bracket models, and clinical trials, none have obtained accurate measurements^52,55^.

Besides orthodontic treatments, the development of accurate force sensors is also needed in several other oral and dental health applications. For example, maximum bite force is a useful metric in addition to specific morphological classification of malocclusion to determine the severity and functional outcome of the disease^56–61^. Another clinical need for force measurement is in dental implants. Strong foundational support of the alveolar bone is essential for the anchoring of an implant^62^. Currently, information regarding bone formation is obtained via radiographic X-ray imaging, which can only be done in a dental clinic and not compatible with continuous monitoring due to radiation exposure. Biosensors integrated with dentures can be used to assess the mechanical fit, stability, and biocompatibility without the use of X-ray imaging^63^. This will enable wireless and continuous implant information collection, which reduces the number of dental visits, is cost-effective, and could potentially improve the efficacy of dental implants.

Temporomandibular disorder, TMD, is classified as a disorder of the temporomandibular joints in the jaw that can be caused by trauma, injury, malocclusion, or different stress factors. This disorder can cause pain, limitations in opening and closing of the mouth, and dysfunction in daily activities such as chewing, affecting up to 12% of the population. Due to communication with the blood, saliva has shown to display elevated biomarkers associated with TMD, such as salivary cortisol, which is indicated as a stress biomarker for the disorder^64^. Other studies have found higher levels of salivary and plasma glutamate in patients with TMD, suggesting that the dysregulation of glutamate may be involved in the pathophysiology of TMD^65^. In some studies, higher levels of IL-1 have also been found in patients with TMD alone, showing a relationship between inflammatory mediators and the disorder^66^. Saliva has shown to be a non-invasive potential biomarker for TMD; however, continuous monitoring systems have yet to be developed to monitor the disorder. Literature suggests a developing need for biosensors related to the treatment of TMD.

### Bruxism

Bruxism is a parafunctional habit in which individuals subconsciously clench their jaw for extended periods of time during their sleep. This habit can result in worn dentition, dysfunction of the temporomandibular joint, pain, and tooth sensitivity. Studies have found that bruxers display higher levels of perceived psychological stress and salivary cortisol levels^67^. The development of an intraoral continuous monitoring system for the detection of bruxism can help clinicians improve patient outcomes.

### Technical challenges

Current medical technology cannot meet the clinical needs due to several technical challenges. First, it is difficult to achieve analytical sensitivity in the physiological range of oral biomarkers under continuous sensor operation. The physiological range of several important biomarkers is very low, while the sample matrix is highly complex. Continuous sensors like continuous glucose monitoring are only available for single analyte and does not work for low-abundance analytes such as cytokines. Second, it is difficult to achieve continuous measurement. Standard immunoassays such as ELISA can precisely quantify the concentration of the analyte. However, the necessity for large assay volumes, manual sampling intervals, and prolonged incubation periods limits the frequency of measurements and introduces delays that prevent continuous measurement. Third, the dynamically changing oral environment makes anti-biofouling difficult. The oral environment can dynamically change depending on oral hygiene, food intake, and other physiological states^68,69^. This presents unique challenges for the device design of biosensors in the oral cavity. Independent of the signal-transduction mechanism, sensor specificity and selectivity are often dependent on the probe–target interaction and the type of surface passivation employed. Biofouling, such as nonspecific adsorption of random targets and matrix contaminants onto the sensor surface, instability of the immobilized probe, and detachment of the passivation layers, can degrade the sensor specificity and sensitivity and cause signal drift over time. Preventing nonspecific adsorption while withstanding the constantly changing environment is especially essential for developing sensors for dental and oral applications in this complex environment^70,71^. At the same time, the sensing device needs to be in a minimally invasive, compact format with a sensing probe, data readout, battery, and wireless transmission all integrated. All of these requirements highlight the engineering challenges of interfacing a sensing device in the oral cavity^53^. Next, we review recent developments in biosensing technologies targeting oral and dental healthcare applications.

## Sensors for biochemical monitoring in oral health applications

### Bioanalytical sensor design and characterization

**Fig. 2:**
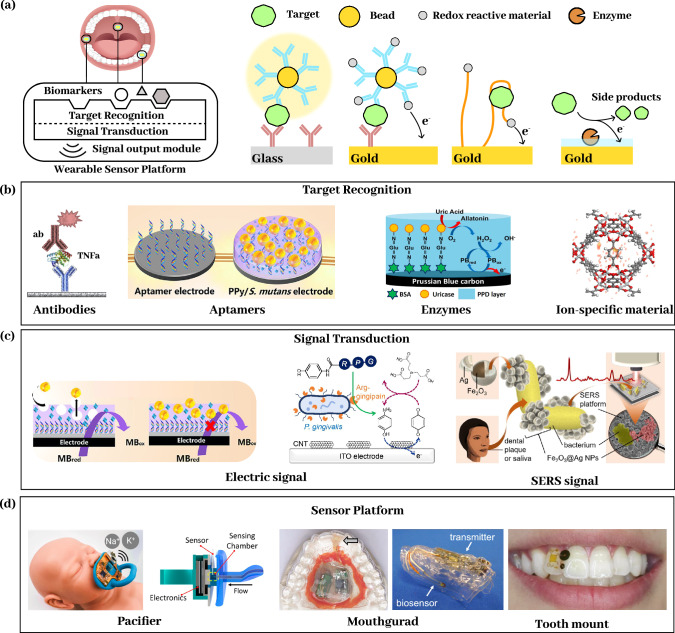
Components of wearable biochemical sensors. **a** Overview of biochemical sensor design, components, and working principles. **b** The target recognition elements, such as antibodies^221^, aptamers^138^, enzymes^10^, and ion-specific material^156^ can specifically interact with the analytes through binding/enzymatic reactions. **c** The signal transduction module converts the molecular reaction events into a signal that can be measured, such as electrical and optical signals^138–140^. **d** Examples of sensor platforms for oral applications include pacifiers^16^, mouthguards^11,164^, and tooth‑mounted devices^8^.

Bioanalytical sensors are typically composed of three core components (Fig. 2a): a bio-recognition element that selectively interacts with target analytes^72^ (Fig. 2b), a signal transduction element that converts the biochemical interaction into a measurable signal (Fig. 2c), and a signal readout element that records or displays the processed output^73–77^. The bio-recognition elements comprise categories of enzymes^11,13,14,17,78^, target selective materials^16,79–82^, and binding reagents such as aptamers^83–86^, antibodies^87–92^, or synthetic binders^15,93^. Biorecognition signals are commonly transduced and observed either optically by imaging photon changes or electrically by probing electron transfer. Adapting these systems for wearable oral applications requires integration of all components into a compact, biocompatible, and mechanically robust sensor platform capable of maintaining stability and performance under physiological conditions^73^ (Fig. 2d). Current intraoral biosensor platforms have been integrated into a variety of device formats such as dentures, mouthguards, modified wooden tongue depressors, dental floss, and toothbrushes. Antifouling strategies are employed to allow these platforms to withstand the complex environment, reduce the overtime calibration drift, and prevent nonspecific absorption of complex proteins and contaminations to ensure selectivity and sensitivity. The antifouling strategies include passivation with molecules such as casein^90^, biotin, molecule monolayers^94^, or poly (ethylene glycol) (PEG)^89^, and protecting the sensor through coating a protection layer such as ion-selective membranes^16,79–82^. These platforms are calibrated either in actual or artificial saliva and gingival crevicular fluid matrices to correct for matrix effects. These platforms have been employed to detect a broad range of clinically relevant biomarkers. For oral and dental applications, common analytes include glucose, nitrite, lactate, uric acid, thiocyanate, sodium, and potassium^95^. Additionally, several protein biomarkers, such as MMP-8, CRP, TNF-α, and IL-6, have shown utility in diagnosing and monitoring periodontal diseases^25^. In this section, we review continuous biochemical sensors designed for biomarker monitoring in dental and oral health applications. To evaluate and compare the analytical and device performance, we will assess dynamic range, sensitivity, specificity, temporal resolution, and longevity as key metrics for assay development. Table 2 summarizes and compares the principal types of bioanalytical sensors for dental, oral, and craniofacial applications.

**Table 2:** Bioanalytical sensors for intra-oral application

Sensor types	Target range	Dynamic range	Longevity	Anti-biofouling	Ref
Electrochemical biosensor	Cytokines, other proteins, bacteria, small molecules	10^−12^–10^−8^ M	Several Hours	Passivation molecules	Table 4, Table 9
Surface plasmon resonance sensor	Cytokines	10^−12^–10^−10 ^M	Several Hours	Passivation molecules	Table 4
Ion-selective electrode	Ions	10^−3^–1 M	Several Days	Ion-selective membranes	Table 7
Enzymatic biosensors	Small molecules, ions, bacteria	10^−5^–10^−3 ^M	1 h	-	Table 7, Table 8
Fiber optic biosensor	Cytokines	10^−14^–10^−7 ^M	-	Passivation molecules	Table 4
Electrochemiluminescence	Cytokines	10^−15^–10^−8 ^M	1 h	-	Table 4

### Cytokine monitoring

Cytokines, including IL-1b, IL-6, IL-8, IL-17, and TNF-α, are important biomarkers for oral disease such as gingivitis, periodontitis, and oral cancers^96–101^. Previous studies have shown that concentrations of IL-6, IL-8, and IL-17 are elevated in patients with chronic periodontitis compared to periodontally healthy individuals^96,102^. For OSCC, salivary levels of IL-6 and IL-8 are significantly higher in patients compared to control populations^97,103^. Another study reports that severe gingivitis patients exhibit significantly elevated salivary IL-6, IL-1β, and CRP compared to controls^104^. Gingival crevicular fluid and salivary cytokine concentrations are summarized in Table 3. Alterations in cytokine biomarkers reflect disease status and progression. Precise quantification enables dentists to intervene before the disease advances to a severe stage.

**Table 3:** GCF and salivary cytokines levels

Biomarker	Disease	Control	Patients	Matrix	Ref
IL-6					
	Periodontitis	0.28 ± 0.16 pM	3.0 ± 2.4 pM	Saliva	^96^
			0.42 ± 0.01 pM	GCF	^218^
	OSCC	0.62 ± 0.16 pM	3.55 ± 0.70 pM	Saliva	^103^
	Gingivitis	0.48 pM	0.75 pM	Saliva	^104^
		6.6 pM	19 pM	GCF	^219^
IL-8					
	Periodontitis	33 ± 13 pM	116 ± 75 pM	Saliva	^102^
			4.25 ± 0.14 pM	GCF	^218^
	OSCC	31.3 pM	90 pM	Saliva	^97^
IL-1β					
	Gingivitis	0.95 pM	1.75 pM	Saliva	^104^
IL-17					
	Periodontitis	34.6 ± 26.3 pM	481 ± 267 pM	Saliva	^96^
TNF-a					
	Periodontitis		1.2 ± 0.03 pM	GCF	^218^

**Fig. 3:**
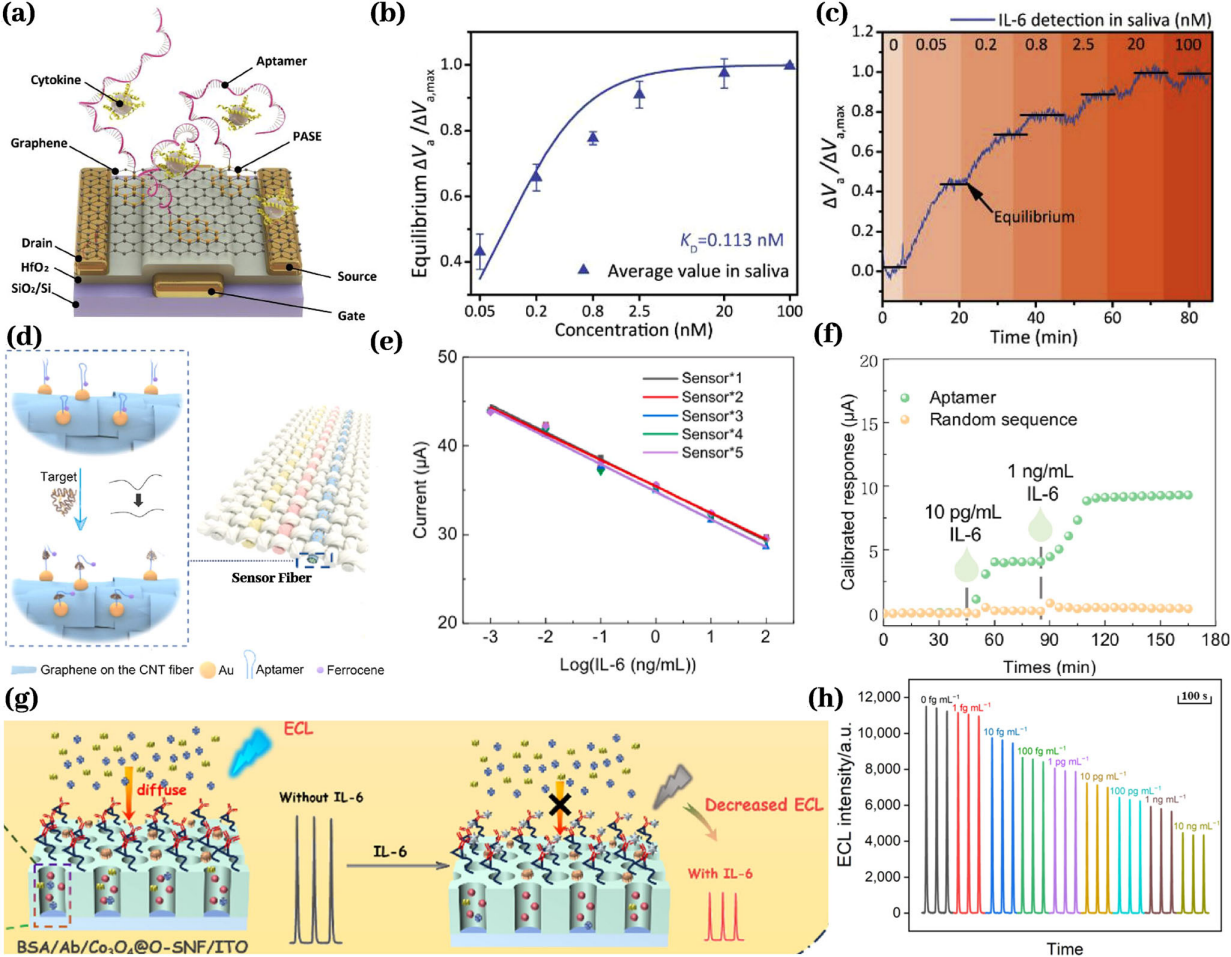
Wearable continuous cytokine biosensors. **a** An aptamer-based field effect transistor biosensors responds to the change of IL-6 concentration within 400 s in saliva with a detection range from 50 pm to 100 nM (**b**) for 80 mins in saliva (**c**)^85^. **d** Another novel electrochemical fabric based on aptamer-functionalized carbon nanotube/graphene fibers could detect IL-6 in a range from 1 pg/ml to 100 ng/ml **(e)** for 3 h (**f**)^86^. **g** An IL-6 sensor demonstrated continuous monitoring from 1 fg/mL to 10 ng/mL for 60 min in GCF (**h)**^105^.

Recent studies have explored continuous biochemical sensors capable of monitoring the aforementioned pro-inflammatory cytokines in biofluids, including saliva^83,85,87–92^. While a few wearable sensors have been designed to continuously track cytokines^84,86^ in sweat and other peripheral biofluids, no wearable platforms have yet been successfully implemented for continuous cytokine detection in the oral cavity. Nonetheless, several existing cytokine biosensors exhibit design features and performance specifications that could be engineered and adapted for intraoral use. Cytokine biosensors primarily employ antibodies^87–92^ or aptamers^83–86^ as their biorecognition elements. Another biosensor employs complementary oligonucleotides to quantify IL-8 mRNA^92^. These sensors typically transduce signals via electrochemical^83,85–87^ or optical^89,91^ methods. Electrochemical cytokine sensors employ potentiometric transducers, such as field-effect transistors^85,87^ (Fig. 3a–c), to measure electrode potential differences, and amperometric transducers to quantify reaction currents at a fixed potential^83,86^. Electrochemical transduction enables facile miniaturization and provides biocompatible, stable electrode interfaces. Optical cytokine biosensors, such as SPR platforms in a study^89,91^, detect IL-8 by monitoring resonance shifts when the analyte binds to an anti-IL-8 coated surface. Optical biosensors typically depend on bulky instrumentation, hindering their miniaturization into intraoral wearable formats. No intraoral wearable cytokine biosensor has been reported, although promising formats for cytokine detection in other biofluids have been proposed. A recent study integrated electrochemical fibrous sensors into wristband and headband formats to monitor IL-6 in sweat^86^ (Fig. 3d–f). Another implanted aptamer-based sensor employing oscillation-driven active reset and electrochemical transduction quantified IL-6 and TNF-α in interstitial fluid^84^. Wearable cytokine sensors have demonstrated effective recognition, signal transduction, and operational durability in non-oral contexts, indicating their potential for adaptation to intraoral formats. An IL-6 sensor was created by modifying a nanochannel film with a catalyst and antibodies. The sensor was built on an ITO electrode using a straightforward growth method. Its surface was functionalized to attach capture antibodies, creating a specific detection interface for IL-6 under neutral conditions. IL-6 binding to this interface changed the sensor’s electrical signal, enabling precise measurement of IL-6 levels within 1 fg/mL to 10 ng/mL^105^ (Fig. 3g, h).

Most electrochemical and optical cytokine biosensors achieve detection limits from low pg/mL to ng/mL, encompassing physiological cytokine concentrations. By employing target-specific aptamers or antibodies, these cytokine sensors achieve high specificity. Nearly all continuous cytokine biosensors yield only rising signals, as slow target dissociation and the absence of active reset mechanisms prevent real-time tracking of dynamic concentration changes^84^. Table 4 summarizes the performance metrics of the cytokine sensors. Interestingly, an electrochemiluminescence (ECL) biosensor for IL-6 achieved attomolar-to-picomolar sensitivity in gingival crevicular fluid, but its reliance on bulky instrumentation prevents miniaturization for wearable oral applications^105^. Several continuous cytokine biosensors have demonstrated accurate target monitoring over multi-hour durations^85,86,89^. In one study, the wearable sweat-based IL-6 sensor retained performance after roughly 1000 use cycles without significant degradation^86^. Continuous cytokine tracking has been realized in saliva and gingival crevicular fluid in non-intraoral setting using electrochemical and optical biosensor platforms. To date, no intraoral wearable has been reported that can capture cytokines in real time.

**Table 4:** Biosensor for cytokines

Analyte	Recognition element	Readout signal	Format	Matrix	Dynamic range	Longevity	Ref
IL-1b							
	Antibody	Electrical	Single read	Saliva	9.2 × 10^−^^4^ – 2.9 × 10^−^^2^ pM		^90^
IL-6							
	Aptamer	Electrical	Continuous	Saliva	5 × 10^1^ – 10^5^ pM	80 min	^85^
	Aptamer	Electrical	Wearable	Sweat	5 × 10^−2^ – 5 × 10^3^ pM	180 min	^86^
	Antibody	Electrical	Single read	GCF	4.8 × 10^−2^ – 4.8 × 10^3^ pM		^220^
	Antibody	ECL	Continuous	GCF	4.8 × 10^−5^ – 4.8 × 10^2^ pM	60 min	^105^
	Aptamer	Electrical	Wearable	ISF	0.48 – 23.8 pM	750 min	^84^
IL-8							
	Antibody	Optical, SPR	Continuous	Buffer	9.5 – 191 pM		^91^
	Antibody	Electrical	Single read	Saliva	29 – 595 pM		^92^
TNF-a							
	Antibody	Electrical	Single read	Saliva	5.9 × 10^−2^ – 1.76 pM		^88^
	Aptamer	Electrical	Single read	Buffer	10^−2^ – 10^3^ pM		^83^

Future advancements will demand miniature, robust electrochemical or optical transducers designed for the oral cavity, biocompatible anti-fouling coatings to reduce saliva-induced drift, and low-power wireless telemetry for uninterrupted data acquisition.

### Quantifying other protein analytes

Non-cytokine salivary and GCF proteins such as MMPs, ALP, Osteoprotegerin (OPG), and C-reactive protein (CRP) also serve as key oral health biomarkers^106–110^ (Table 5). Levels of enzymes such as MMPs (e.g., MMP-8 and MMP-9) and ALP are significantly elevated in GCF and saliva of chronic periodontitis patients compared to healthy individuals^111–114^. Elevated levels of MMPs, particularly MMP-2, MMP-9, and MMP-12, are frequently observed in oral cancer and are associated with tumor progression and metastasis^115,116^. OPG is markedly lower in chronic periodontitis than in healthy individuals^117^, whereas CRP levels increase stepwise from health to gingivitis to periodontitis^118^. Besides, children with reduced salivary α-amylase (sAA) activity exhibit increased susceptibility to dental caries^119^. Timely detection of non-cytokine protein biomarkers informs oral disease severity and progression, enabling early intervention.

**Table 5:** Salivary levels of non-cytokine proteins

Biomarker	Disease	Control	Patients	Ref
MMP-8				
	Periodontitis		3.8 nM	^112^
	Gingivitis		1.8 nM	^112^
MMP-9				
	Periodontitis	1.04 ± 0.09 nM	2.18 ± 0.41 nM	^113^
CRP				
	Periodontitis	27.0 ± 31.1 nM	46 ± 44 nM	^118^
	Gingivitis	27.0 ± 31.1 nM	30.8 ± 26.6 nM	^118^
MMP-12				
	OSCC	~20 nM	~19.1 nM	^115^
OPG				
	Periodontitis	1.7 ± 0.3 pM	0.92 ± 0.30 pM	^117^
ALP				
	Periodontitis	<0.33 pM	>0.33 pM	^111^

Many biosensors have been developed to quantify non-cytokine salivary and GCF proteins implicated in oral and dental health^120–129^. Most current biosensors are configured for single measurements, although certain designs could be adapted for continuous monitoring and wearable integration. Non-cytokine protein biosensors rely on antibody-based affinity recognition^123–126,128,129^ or enzymatic reaction-based recognition^122,127^ mechanisms. Electrochemical transduction predominates among these biosensors, with optical and acoustic methods also employed for signal transduction.

In an rGO-based electrochemical sensor, MMP-7 cleaves surface-bound peptides, reducing their mass and charge and thus lowering the measured current^127^. In another ALP sensor, ALP catalyzes phenyl phosphate hydrolysis to phenol, which is quantified by square-wave voltammetry^122^. In another example, an ultrasensitive electrochemical immunoassay achieved rapid, simultaneous IL-6 and MMP-9 detection on a heated screen-printed carbon electrode (HSPCE) modified with hydrophilic, low-toxicity graphene nanoribbons for antibody immobilization and signal amplification. PS@PDA-metal nanocomposites labeled the detection antibodies, producing a strong voltametric response in acetic buffer. A sandwich format on adjacent HSPCE electrodes eliminated crosstalk and enabled quantification from 10⁻⁵ to 10³ ng/mL^124^.

These sensors span hundreds of pg/mL to hundreds of ng/mL, matching salivary and GCF protein levels observed in oral disease. Specificity is achieved using target-specific antibodies or substrate-selective enzymatic reactions. The enzymatic reaction-based biosensors are unsuited for continuous operation because substrate turnover depletes surface-bound reagents and demands external reagent supply, precluding uninterrupted monitoring. Affinity-based electrochemical protein biosensors preserve surface-bound biorecognition elements after each assay, enabling continuous monitoring and integration into wearable formats. Extending these biosensors to continuous operation requires enhanced longevity; applying advanced surface passivation can mitigate degradation and enable prolonged monitoring^94,130^.

### Identification of infection

Oral bacteria critically influence dental and periodontal health by driving biofilm formation, acid production, and inflammatory responses^131–135^. Clinical studies reveal that *S. mutans* predominates in individuals with dental caries, while Streptococcus sanguinis levels decline^131,136^. *P. gingivalis* abundance is markedly elevated in periodontitis patients(10^5^–10^8^ CFU/mL) compared to healthy individuals^137^. Other bacteria/pathogen such as Actinomyces species, *Fusobacterium nucleatum*, and *T. denticola*—contribute to plaque maturation and tissue destruction. These microbial-level changes highlight the need for biosensors capable of sensitive, species-specific detection in saliva or crevicular fluid. Such devices must discriminate closely related bacteria, operate across clinically relevant concentration ranges, and ideally support multiplexed, real-time monitoring to enable early diagnosis and guide personalized oral care.

**Fig. 4:**
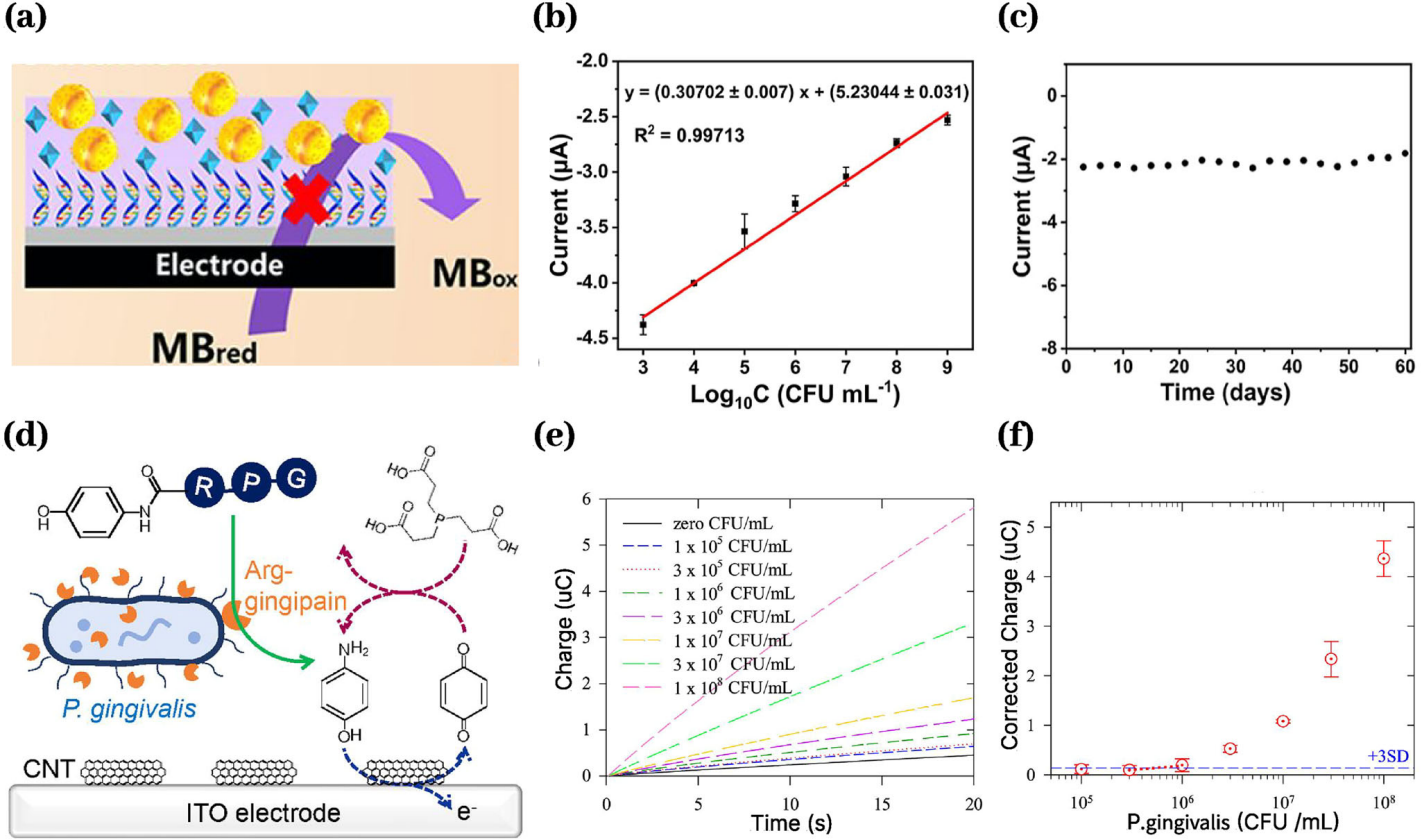
Continuous/Wearable bacteria biosensors. **a** A portable saliva sensor based on aptamer and *S. mutans*-imprinted polymers (SIPs) demonstrated measurement of *S. mutans* from 10^3^–10^9 ^CFU/mL (**b**) and is stable for 60 days (**c**)^138^. **d** A washing- and separation-free electrochemical sensor demonstrated indirect detection of *P. Gingivalis* in saliva from a range 10^5^–10^8^ CFU/mL **e**, **f**^139^.

Continuous and end-point biosensors have been developed for salivary bacteria and pathogen detection^93,138–143^, and some wearable devices enable intraoral microbial monitoring^15^. Bacterial biosensors employ affinity-based recognition elements such as aptamers^138^, antibodies^141,142^, antimicrobial peptide (AMP)^15,93^, and molecularly imprinted polymers (MIP)^144^ or an indirect detection method that monitors bacteria-mediated reactions^143^. These bacterial biosensors predominantly employ electrochemical transduction. A biosensor integrates an *S. mutans* specific DNA aptamer and MIP on an electrode, using methylene blue as a redox probe. Binding of *S. mutans* occludes electron transfer between methylene blue and the electrode, causing a current decrease proportional to bacterial concentration. It quantifies *S. mutans* across the clinically relevant range for high caries risk and stable for about 60 days^138^ (Fig. 4a–c). A wash-free electrochemical assay for *P. gingivalis* uses glycine-proline-arginine-4-aminophenol (with glycylglycine boosting Arg-gingipain activity) as the proteolytic substrate. Released 4-aminophenol undergoes EC redox cycling with tris(2-carboxyethyl)phosphine, and matrix-corrected charge differences before and after a 15-min incubation yield high, reproducible, pathogen-specific signals in saliva^139^ (Fig. 4d–f). Indirect assays requiring exogenous substrates preclude continuous or wearable implementation due to the need for reagent replenishment. A graphene sensor functionalized with self-assembled antimicrobial peptides enables bio-selective, single-cell bacterial detection and is powered wirelessly via an integrated resonant coil, eliminating onboard batteries or wiring. Printed on water-soluble silk, it can be bio-transferred onto tooth enamel for seamless intraoral integration. It demonstrated bacterial detection in seconds across at least five consecutive cycles^15^. By interchanging the surface affinity element, the platform can be tailored to detect *S. mutans*, S. sanguinis, or *P. gingivalis* in dental applications.

Reported bacterial biosensors typically span 10^2^–10^9^ CFU/mL, covering physiological salivary pathogen concentrations^15,138,141,142^. Aptamer- and antibody-based sensors achieve high pathogen specificity, whereas antimicrobial peptide–based sensors lack species-level discrimination due to semi-selective binding of AMP^15^. Performance metrics for these biosensors are summarized in Table 6.

**Table 6:** Salivary bacterial biosensors

Analyte	Recognition element	Readout signal	Format	Matrix	Dynamic range	Ref
*S. mutans*						
	Aptamer	Electrical	Single read	Saliva	10^2^ ~ 10^8^ cfu/mL	^138^
	Antibody	Electrical	Single read	Buffer	10^3^ ~ 10^10^ cfu/mL	^141^
*S. sanguinis*						
	AMP	Optical	Single read	Saliva	10 ~ 10^6^ cfu/mL	^93^
*P. gingivalis*						
	Enzyme	Electrical	Single read	Saliva	10 ~ 10^5^ cfu/mL	^139^
	Antibody	Optical	Single read	Saliva	10 ~ 10^7^ cfu/mL	^142^
	Nanomaterial	Optical	Single read	Saliva	10^3^ ~ 10^10^ cfu/mL	^140^
*S. aureus*						
	AMP	Electrical	Wearable	Saliva	10^3^ ~ 10^8^ cfu/mL	^15^

### Biosensor for ions

Ion and metabolite concentrations in saliva and gingival crevicular fluid regulate enamel mineralization, microbial balance, and inflammatory responses, making them critical oral health biomarkers^145,146^. Clinical studies have shown that periodontitis patients exhibit higher salivary sodium, potassium, calcium, and uric acid than healthy individuals^147–149^. In caries prevention, maintaining salivary fluoride^150–152^ within a therapeutic window and adequate calcium^153^ and phosphate^154^ levels supports enamel repair. Altered trace elements—copper, zinc, lead, cadmium, calcium, and magnesium—have been associated with oral potentially malignant disorders and OSCC^155^. These observations underscore the need for biosensors offering selective ion and metabolite detection across physiological ranges, robust performance in complex biofluids, and multiplexed or continuous real-time monitoring.

**Fig. 5:**
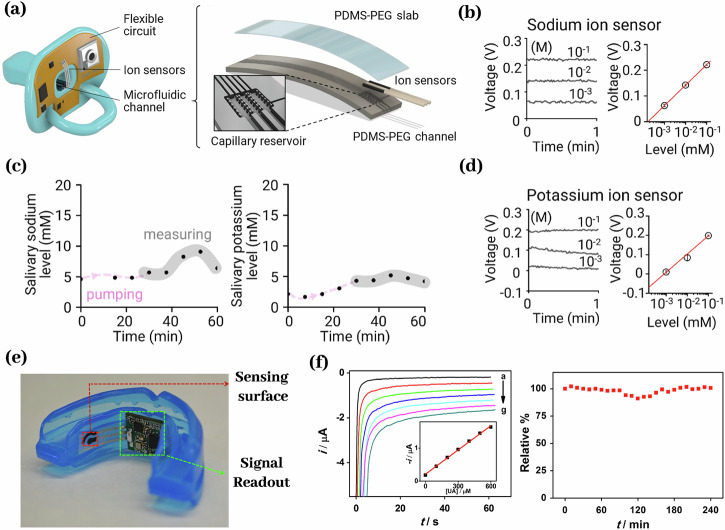
Wearable/continuous ion sensors. **a** An ion-selective electrode-based pacifier sensor could measure sodium **b** and potassium (**c**) from 10^−3^–10^−1 ^M for 1 week (**d**)^16^. **e** An enzyme-based mouth guard sensor could measure uric acid from 0 to 1 mM and could last for 4 days (**f**)^10^.

Multiple biosensors have been reported measuring salivary ions such as sodium^16,81,82^, potassium^16^, uric acid^10,156^, fluoride^79^, and phosphate^157^. Wearable sensors are already available for sodium^81,82^, potassium^16^, and uric acid^10^, continuous sensors are available for fluoride^79^ and phosphate^158^. Wearable salivary ion sensors predominantly utilize ion selective electrodes^16,79–82^, with some designs based on enzymatic or catalytic reactions^10,157^ and others incorporating ion selective nanomaterials^156^. Most of these sensors employ electrochemical transduction. An ISE biosensor quantifies Na⁺ or K⁺ activity via a selective membrane that generates a potential, proportional to the logarithm of ion activity, measured against a reference electrode. Its ion-selective membrane also serves as an antifouling barrier, protecting the electrode and maintaining stability for up to 1 week^16^ (Fig. 5a–d). An enzymatic electrochemical uric acid sensor catalyzes UA oxidation to modulate electron flux at the electrode, generating a current proportional to UA concentration. A phenylenediamine membrane provides antifouling protection and sustains stable performance for up to 4 days^10^ (Fig. 5e, f). A SERS based salivary UA sensor employs Au/MIL-125(Ti) nanocomposites that selectively adsorb UA via π–π stacking with benzene rings of MIL-125(Ti), enabling rapid, sensitive detection^156^.

ISE sensors detect sodium and potassium from 10⁻⁴ to 1 M and fluoride from 10⁻⁶ to 10⁻² M^79^, while the UA sensor spans 0–1000 µM^10^, fully covering physiological salivary ranges. By using ion selective membranes, nanocomposite adsorption, or enzyme catalyzed reactions, these sensors achieve high specificity for their target ions. However, ISE sensors can exhibit signal crosstalk when non target ions permeate the selective membrane^81^. These ion sensors provide rapid responses, typically within tens of seconds. Their stability and facile miniaturization enable integration into intraoral wearables such as mouthguards^10–13,79,81^, tooth mounted films^14,15^, and pacifiers^16,17^. Performance metrics for these biosensors are summarized in Table 7.

**Table 7:** Salivary ion sensors

Analyte	Recognition element	Readout signal	Format	Matrix	Dynamic range	Longevity	Ref
Na							
	ISE	Electrical	Wearable	Saliva	10^−3^ – 1 M	1 week	^81^
	ISE	Electrical	Continuous	Saliva	10^−8^ – 10^−3^ M		^82^
	ISE	Electrical	Wearable	Saliva	10^−3^ – 10^−1^ M	1 week	^16^
K							
	ISE	Electrical	Wearable	Saliva	10^−3^ – 10^−1^ M	1 week	^16^
UA							
	Enzyme	Electrical	Wearable	Saliva	0 – 10^−3^ M	4 days	^10^
	Nanomaterial	Optical	Continuous	Saliva	5 × 10^−5^ to 5 × 10^−3^ M		^156^
F							
	ISE	Electrical	Continuous	Saliva	10^−2^ – 10^−6^ M	1 h	^79^
PO4							
	Enzyme	Electrical	Single read	Saliva	1.6 × 10^−4^ ~ 2 × 10^−3^ M		^157^
	Enzyme	Electrical	Continuous	Saliva	7.5 × 10^−6^ ~ 6.25 × 10^−4^ M	12 h	^158^

### Small molecule detection

Salivary glucose and lactate critically influence oral health by modulating immune defenses, biofilm acidity, and tissue integrity. In diabetic periodontitis, salivary glucose rises above physiological levels, impairing leukocyte chemotaxis, thickening the vascular basement membrane, and weakening periodontal defenses^159^, while also fueling lactic acid production by oral biofilms. Elevated salivary lactate further lowers plaque pH, accelerating enamel demineralization and caries^160,161^. These pathological elevations—relative to normal saliva glucose (0.5–1.0 mg/100 mL) and lactate (0.1–2.5 mmol/L)—underscore the need for biosensors with micromolar-to-millimolar sensitivity, robust anti-fouling design, and real-time readout capability to detect and track metabolic shifts in saliva^162^.

**Fig. 6:**
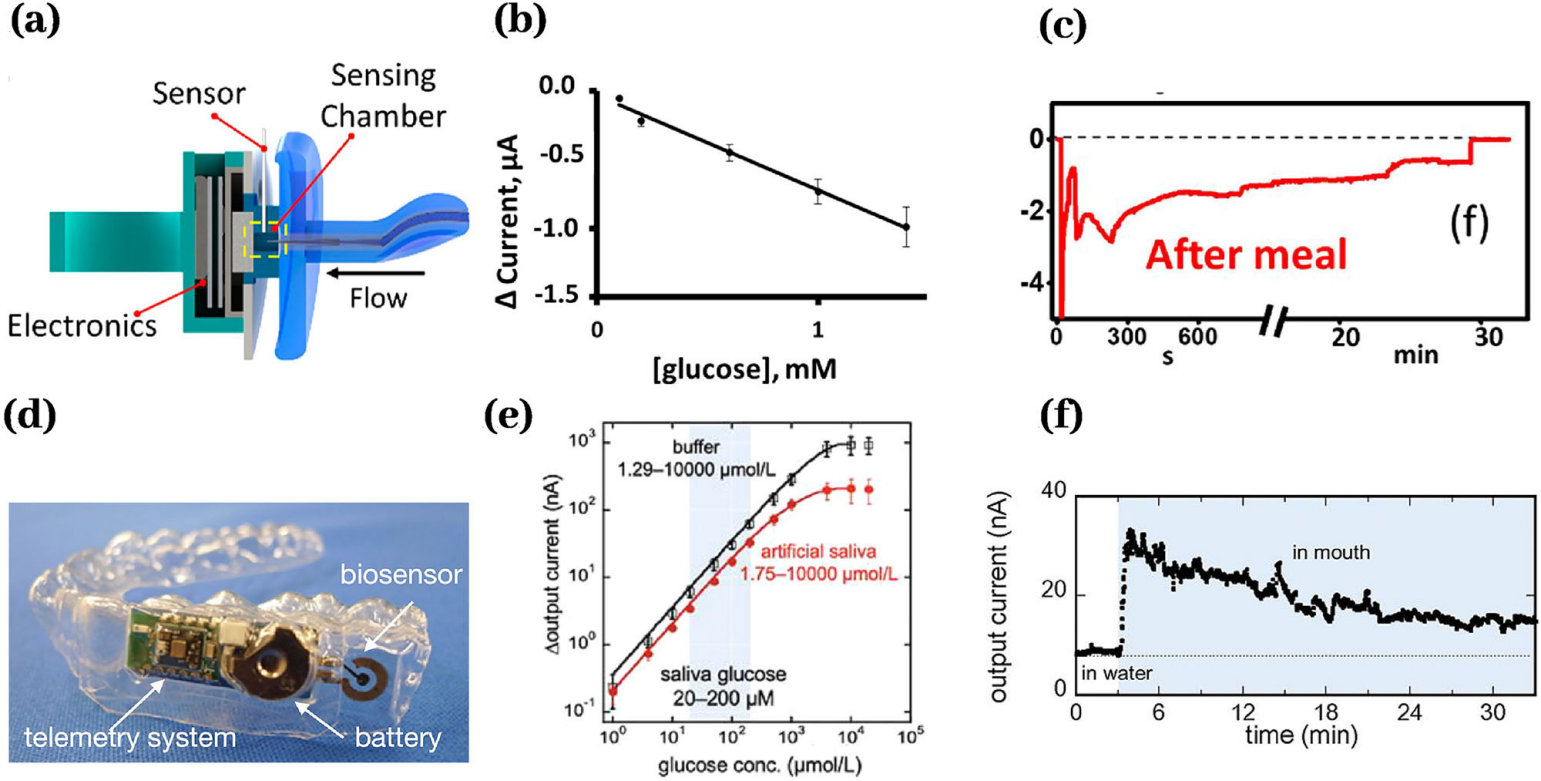
Wearable biosensors for glucose. **a** A pacifier biosensor measures glucose from 0.1 to 1.4 mM (**b**) for 30 min (**c**)^17^. **d** A mouthguard biosensor measures glucose from 1.75 to 10,000 µmol/L (**e**) for more than 30 min (**f**)^12^.

Multiple wearable biosensors have been reported measuring salivary glucose^11,13,14,17,78^ and lactate^12,162^. Most Glucose and lactate biosensors rely on enzyme-catalyzed recognition coupled with electrochemical transduction. In an enzymatic electrochemical glucose sensor, an electrode immobilized glucose oxidase (GOx) oxidizes glucose to gluconolactone and hydrogen peroxide, and the subsequent electrooxidation of hydrogen peroxide at the electrode produces a current proportional to glucose concentration^11,13,17,78^ (Fig. 6). A mouthguard lactate sensor immobilizes lactate oxidase on the working electrode to catalyze lactate oxidation, generating an electrochemical current proportional to lactate concentration^12^. In another study, a materials-based strategy embeds an active sensing layer between two reverse-facing split-ring resonators in a compact RF construct, enabling glucose detection via shifts in resonant frequency and amplitude; adding tailored functional coatings could impart molecular specificity. Although this method lacks molecular specificity, tailored functional coatings could be introduced to enable selective analyte binding^14^.

Enzymatic electrochemical glucose sensors operate over 1–10,000 μM and lactate sensors over 0.1–1.0 mM, covering physiological salivary concentrations. Specificity arises from enzyme–substrate recognition, with immobilized enzymes remaining active for repeated measurements. These electrochemical sensors respond within seconds and retain functional stability for over 30 min. Electrodes are miniaturized and coated with a poly-ortho-phenylenediamine antifouling film, then integrated into wearable platforms such as pacifiers^17^, mouthguards^11–13^, and tooth-mounts^14^. The performance metrics for these biosensors are summarized in Table 8.

**Table 8:** Glucose and lactate biosensors

Analyte	Recognition element	Readout signal	Format	Matrix	Dynamic range	Longevity	Ref
Glucose							
	Enzyme	Electrical	Wearable	Saliva	5–10^3^ mM		^11^
	Enzyme	Electrical	Wearable	Saliva	0.1–1.4 mM	30 min	^17^
	Enzyme	Electrical	Wearable	Saliva	1.75 × 10^−3^ – 10 mM	30 min	^13^
		Radiofrequency	Wearable	Saliva	11–55 mM	30 min	^14^
	Enzyme	Electrical	Wearable	Saliva	10^−5^–1 mM		^78^
Lactate							
	Enzyme	Electrical	Wearable	Saliva	0.1–1 mM	2 h	^12^
	Enzyme	Electrical	Continuous	Saliva	0.1–2.5 mM		^162^

### Biosensor for pH

The pH homeostasis within the body can impact metabolic reactions as well as biological transport systems, which can manifest within the oral cavity^163^. In normal situations, human pH levels range from 6.6 to 7.1, and salivary pH levels that deviate from the norm are indicative of physiological diseases^164,165^. Low pH levels of one’s oral microbiome indicate a strong relationship between increased caries risk, enamel demineralization, and gingival inflammation^166^. pH levels in the oral cavity can also affect the quality and strength of dentures in a patient’s mouth. Decreased oral pH may be caused by everyday activities such as sugar intake, which promotes acid production and bacterial accumulation, or health conditions such as gastroesophageal reflux^167^. Degradation of chemical structures within the denture material can be amplified in an acidic environment, leading to a rough denture surface, cracks, and inability to resist fractures^167^. Examining pH levels in the human body can provide insights into occurrences in the oral cavity and digestive tract. These insights can help the provider make informed decisions when choosing the most appropriate denture material for each patient, as well as monitoring their oral health status.

Several electrochemical-based salivary pH sensors have been reported, utilizing diverse transduction principles. A potentiometric pH sensor employing a thin antimony electrode interacts with hydrogen ions to generate a potential proportional to pH, maintaining accurate measurements over pH 1–9 for 24 h. A conductimetric pH sensor employs a polyaniline membrane whose protonation and deprotonation modulate its conductivity, with pH inferred from the resulting resistance changes^168^. A mouthguard-based voltammetric pH sensor uses proton-selective redox probes anchored to multiwalled carbon nanotubes to convert pH shifts from 2 to 11 into electrical signals^169^. Current pH sensors are highly mature that deliver rapid (second-scale) responses across pH 2–11, encompassing the full salivary range, and maintain stable performance for over 20 h.

## Sensor for dental force measurement

Continuous monitoring of forces is essential for delivering personalized and biologically optimized orthodontic treatment^170^. A major technical challenge is to capture complex, dynamic forces acting on teeth in vivo, in real-time, and with high precision. Traditional approaches, including finite element simulations or force gauges on brackets, fail to reflect the temporal and spatial variability of intraoral forces, particularly with clear aligners or removable appliances^171^. To bridge this gap, sensor-integrated systems capable of measuring multidirectional forces continuously in the oral cavity are needed. Typical orthodontic forces vary depending on the type of tooth movement. Tipping and rotational movements generally require 35–60 g (0.343–0.686 N), while bodily movement demands higher forces around 70–120 g (0.686–1.176 N)^172^. More delicate movements like intrusion use as little as 10–20 g (0.098–0.196 N) to avoid root damage. In clear aligner treatments, the average force per tooth typically falls between 20 and 100 g (0.196–0.98 N)^173^, though this may decay over time due to material relaxation and intraoral conditions. These ranges provide approximate benchmarks for the sensitivity and resolution in force sensing systems.

### Force sensor format and components

**Fig. 7:**
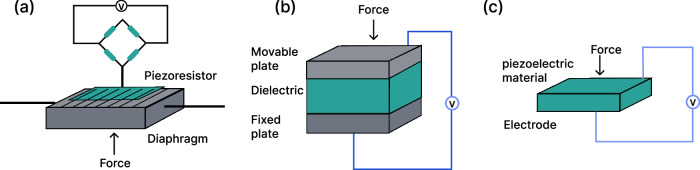
Schematic of different types of force transducer. **a** Piezoresistive sensor detects force by measuring the change in electrical resistance of a material when it is mechanically strained. **b** Capacitive sensor measures force based on changes in capacitance caused by the relative displacement of conductive plates separated by a dielectric layer. **c** Piezoelectric sensor converts mechanical stress into electrical charge using materials that generate voltage when deformed.

Intraoral force sensors typically consist of three core elements: a transducer, a signal processing circuit, and a mechanical interface tailored to the dental anatomy. The transducer converts mechanical forces into readable electrical signals. Typical transducer modalities include piezoresistive, capacitive, or piezoelectric (Fig. 7). These signals are subsequently amplified, filtered, and digitized by a compact electronic device. The mechanical interface packages the force transducer and ensures that intraoral forces are efficiently transferred to the sensing element without compromising patient comfort^174^. To evaluate sensor performance, both analytical and device characteristics must be considered. Analytical performance includes sensitivity, linearity, repeatability, and response time. Sensitivity in orthodontic force sensing typically falls in the range of 20–50 mV/N for piezoresistive sensors, and 5–15% signal change per newton for capacitive sensors, depending on dielectric design^175^. Most oral force sensing systems aim for a minimum detectable force between 10 and 50 mN, which is sufficient to capture subtle differences during orthodontic adjustment or bite evaluation^176^. To ensure accuracy across a broad force range, force sensors often have linearity above 95%, meaning the sensor’s output remains within 5% deviation from an ideal linear response over the entire measurement range, and maintains low hysteresis (<5% of the full-scale output), ensuring consistent signal behavior during loading and unloading cycles^177^. Repeatability is typically reflected in variations below 3% across measurements, and low signal drift with less than 1% per day, which are essential for long-term monitoring in clinical settings. Fast response times, often under 0.5–1 s, are especially important for capturing transient bite forces or real-time orthodontic changes^55^.

#### Piezoresistive force sensor

Piezoresistive force sensors convert mechanical stress into electrical signals via changes in the resistivity of semiconducting or metal elements. The basic principle relies on strain-induced resistance modulation, typically captured using a Wheatstone bridge configuration^178^ (Fig. 7a). This mechanism offers high sensitivity (e.g., 37.79 mV V^−1^ MPa^−1^)^179^ in a compact form factor, making it one of the most widely used modalities in intraoral force sensing^180^.

**Fig. 8:**
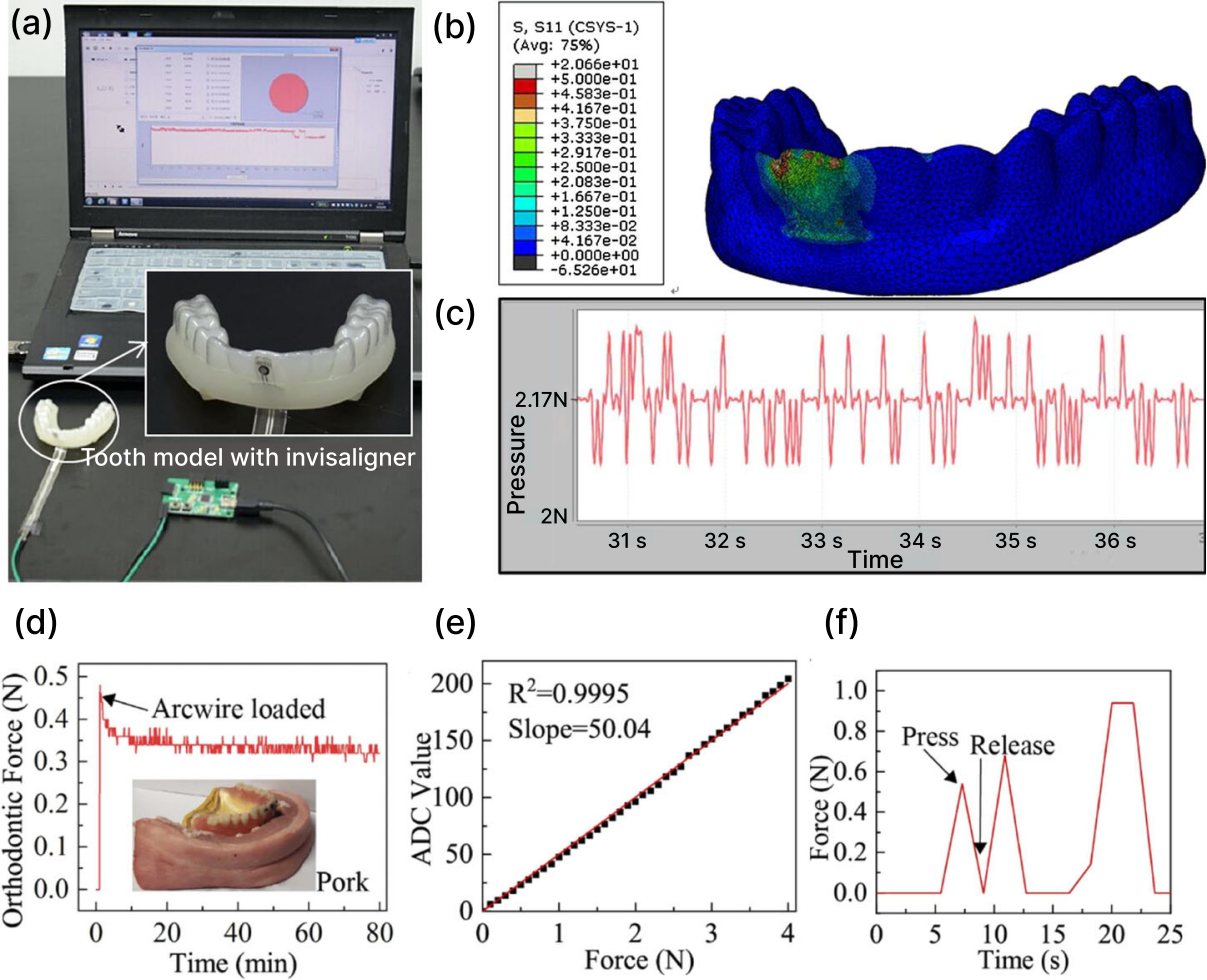
Piezoresistive force sensors. **a**–**c** Force measurement setup in a thin film piezoresistive force sensor^183^. **b** Stress distribution and **c** the measured force changes over time. **d** Measured orthodontic force profile over 80 min in an RF-harvested piezoresistive platform^55^. **e** Correlation of applied force and device readout. **f** Measured force response simulated by finger press.

Several force sensors targeting dental applications have been developed and characterized. Lapatki et al.^181^ developed a “smart bracket” integrating diffused piezoresistors on a silicon die, achieving a force measurement range of ±1.5 N and moment range of ±15 N·mm, with an accuracy of ±0.04 N in force measurement and ±1 N·mm in moment measurement under laboratory conditions. Shi et al. introduced a rosette-type piezoresistive sensor designed for insertion between clear aligners and tooth surfaces^182^. This configuration allows temperature-compensated measurement of the in-plane normal stress difference and the shear stress. Their design featured a 100 μm-thin silicon die and reconstructed forces with a resolution of better than 0.05 N. Minor misalignments or non-uniform tray compression in the design can introduce error margins of up to ±0.04 N, which requires in situ calibration. The system showed excellent linearity and demonstrated clear force stage progression profiles throughout a 7-day aligner wear period. Similar sensor for integration with a clear aligner was also reported by Liu et al.^183^. This sensor was used to quantify the forces of varying thicknesses and activation levels on a resin tooth model. The measured forces were validated against finite element simulation results, showing a standard deviation of 0.2 N (Fig. 8a–c).

Longevity and drift performance are crucial in prolonged intraoral deployment. Li et al. tested a wireless, RF-harvested piezoresistive platform continuously submerged in 37 °C saline over 6 weeks. The sensor-maintained drift within ±3% and sensitivity of approximately 0.13 N across the 0–2 N range (Fig. 8d–f). This study was one of the first to simulate both the biochemical and thermal conditions of the oral cavity over extended durations, providing evidence that piezoresistive sensors can achieve multi-week operational stability without recalibration or rebonding^55^.

#### Capacitive force sensor

Capacitive force sensors operate on the principle that capacitance changes when the applied load perturbs the plate separation, effective area, or the permittivity of the dielectric medium, thus changing the voltage in the bridge circuit composed of capacitors and other resistive elements. (Fig. 7b) Compared to resistive gauges, capacitive sensors have reduced signal noise and better repeatability^184^, highlighting capacitive sensing’s potential to enhance precision in real-time orthodontic monitoring.

**Fig. 9:**
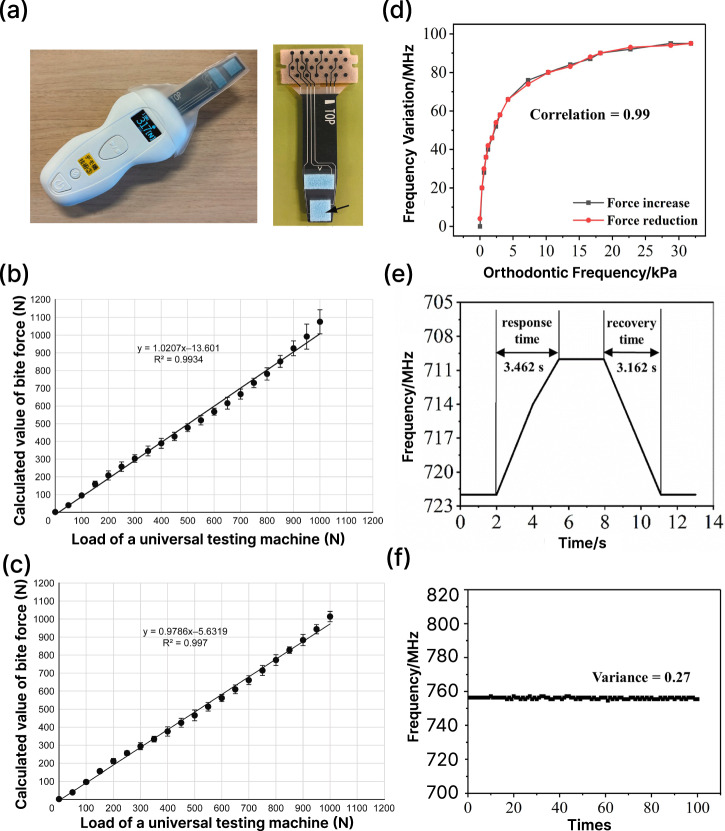
Capacitive sensor performances. **a** The sensor using a capacitive surface pressure distribution^187^. **b**, **c** Calculated results of bite forces for the first and second primary mola, these two calculations suggest that the newly developed capacitive surface pressure distribution sensor can measure occlusal pressure in the first permanent and second primary molars with good reproducibility^187^. **d** Sensor hysteresis^174^. **e** System response time and recovery time^174^. **f** Sensor repeatability, the Variance is 0.27^174^.

Capacitive force sensors have been incorporated in orthodontic force measurements. Hafner et al. integrated four sensors beneath a 0.018” × 0.025” bracket slot and achieved accurate reconstruction of full 3D force (±0.05 N) and moment vectors (±100 N mm)^185^. To improve biocompatibility in the oral environment, Sun et al. present a novel superhydrophobic capacitive sensor with a detection range of up to 400 N^186^. By leveraging a porous and superhydrophobic architecture, the sensor achieves a high sensitivity of 3.2% signal change per kPa, excellent durability (over 15,000 cycle tests), and effective mitigation of biofouling issues^186^. Ogihara et al. developed a sensing device capable of measuring spatially-resolved force, enabling the quantification of pediatric bite force on individual teeth^187^ (Fig. 9a–c). Wang et al. designed an ultra-thin capacitive sensor tailored for clear aligner systems, which use an inductive coil as the transmission unit, a flat capacitor as the signal transducer, and resonance frequency as the readout signal^174^. The sensor exhibited a rapid response time under simulated dynamic conditions (<0.5 s), excellent hysteresis and stability over time^174^ (Fig. 9d–f).

#### Piezoelectric force sensor

When a piezoelectric material is mechanically deformed (compressed, stretched, or sheared), it produces an electric potential across its surfaces that is proportional to the applied force and can be measured as a voltage signal^188^ (Fig. 7c). This type of transducer is highly sensitive and has a fast response time. It is best suited for transient forces measurement, such as bite forces^189^, but has limited responsiveness to low-frequency or static loads, such as orthodontic treatments^190^.

**Fig. 10:**
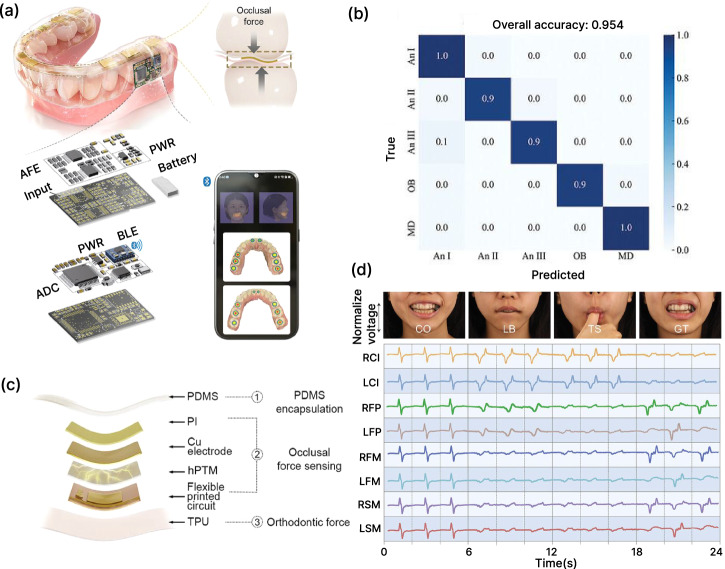
Fully integrated force sensing (ARIA) system^194^. **a** Schematic illustration of ARIA showing the piezoelectric sensor array positioned on the occlusal surface of the teeth, along with the printed circuit board and battery. The exploded view of the board (bottom left) highlights the components, including the analog front-end, analog-to-digital converter, and microcontroller unit. **b** Confusion matrix showing the accuracy of the machine learning model in predicting the malocclusion in the test set. **c** Structural scheme of a 6-layer composite flexible piezoelectric sensor. **d** Photographs of a subject wearing ARIA and performing CO (centric occlusion), LB (lip biting), TS (thumb sucking), and GT (grinding teeth), and the corresponding voltage curve generated during these activities.

An early example of a piezoelectric force transducer in oral application integrates a PVDF-based piezoelectric aligner that sensed forces and also actuated at 30 Hz for potential therapeutic modulation^191,192^. This system differentiated bite and chewing events in the 0–2 N range, with repeatable transduction over multiple cycles. More recently, Hu et al. developed a six-dimensional piezoelectric sensor based on a tenon-and-mortise structure, capable of resolving both force (±1 N) and moment (±4 N mm) across six axes^193^ Their sensor demonstrated multiaxial sensitivity and low hysteresis in simulated orthodontic loading, offering a comprehensive force vector profile that surpasses uniaxial or planar-only devices. For device integration towards clinical use, Feng et al. reported a high-density piezoelectric fiber array into a transparent aligner for real-time monitoring of bite forces and parafunctional activity^194^ (Fig. 10). The highly integrated device in an aligner allows it to monitor bad oral habits, such as lip biting (LB), thumb sucking (TS), and grinding teeth (GT), in addition to detecting the bite status of teeth, as shown in Fig. 10d.

### Clinical applications of force sensors

Unlike static assessments using wax bites or tension gauges, embedded sensors allow dynamic monitoring of force magnitude, direction, and duration in vivo, enabling personalized treatment, early error detection, and remote therapeutic feedback. Their applications broadly fall into two major categories: orthodontic force measurement, which aims to optimize tooth movement by quantifying the forces delivered by brackets or aligners, and bite force measurement, which helps evaluate occlusal function and assess masticatory efficiency in prosthetic and surgical rehabilitation contexts. The clinical utility of these sensors is enhanced by their ability to operate wirelessly and continuously in the complex oral environment. By integrating into aligners, brackets, or occlusal splints, these miniaturized platforms provide clinicians with actionable clinical data that support clinical decision-making.

#### Orthodontic force measurement

Accurately quantify and continuously monitor the three-dimensional loading and temporal adaptation of forces on teeth are of great significance. Studies show that periodontal tissues undergo their most pronounced stress-relaxation within the first 48 h^195,196^ and then level off over the next 7–14 days^196,197^; accordingly, clear aligners themselves are typically replaced every 7–14 days^198^. To achieve continuous force monitoring during this period, several factors such as power consumption, device size and weight, and device robustness in the oral environment need to be considered.

Traditional on-board battery solutions for wearable or implantable systems face limitations in size, longevity, and maintenance. Recent oral biosensor developments have shown energy-efficient architectures and passive communication. Kuhl et al. introduced a battery-free sensor that harvested power via a 13.56 MHz NFC link and streamed data wirelessly at 10 Hz^199^. The device was successfully demonstrated during simulated canine retraction. Similarly, Li et al. reported a piezoresistive platform that harvested RF energy and logged orthodontic forces for over 6 weeks in 37 °C saline, which is enough to span a full wire adjustment interval without requiring sensor replacement or rebonding^55^.

For long-term clinical use, intraoral sensors must be both flexible and miniaturized to ensure comfort and preserve normal oral function. To address this, Lapatki et al. developed a “smart bracket” embedding 32 piezoresistors within a standard twin-bracket footprint, maintaining compatibility with conventional bonding protocols^181^. Kuhl’s subsequent CMOS integration on a 2.9 × 2.9 mm die further pushed miniaturization by embedding 24 stress sensors and an ADC array into a single compact system suitable for clinical integration with brackets^200^. Shi et al. addressed this by back-thinning silicon to just 100 µm, enabling the placement of a rosette-pattern piezoresistive chip between an aligner surface and tooth^182^. The device preserved aligner conformity while enabling accurate 3D force reconstruction. Wang et al. built on this approach by creating an ultra-thin, flexible capacitive film sensor for aligners that provides wireless, real-time, and quantitative force measurements with high positional specificity and sensitivity, supporting clinical comparisons and early detection of staging errors^174^.

The intraoral conditions are characterized by moisture, fluctuating pH, and continuous mechanical stress, which degrade sensor performance over time. To achieve sensor durability in this environment, packaging strategies have evolved from simple silicone coatings to fully hermetic encapsulations using biocompatible polymers. Some systems have demonstrated multi-week operation without signal drift, validating their ability to withstand harsh intraoral conditions^55^.

#### Bite force measurement

A fundamental requirement in bite force sensing is the ability to accommodate the wide range of occlusal forces encountered in vivo. In healthy adults, molar forces typically range from 300 to 600 N^60^, with peak loads exceeding 800 N^201–203^. This places stringent durability demands on sensors, particularly their ability to endure repetitive high-impact compression without calibration drift or mechanical failure. To address this, traditional strain gauge-based devices often utilize metallic forks coated in resilient polymers to buffer occlusal loads and extend device lifespan. However, such rigid designs^204,205^ often causes discomfort and alter natural biting behavior, compromising both patient compliance and reliability for long-term use. To support continuous force monitoring, there is a growing need for flexible, conformable sensors that can withstand the harsh intraoral environment while preserving normal oral function^206,207^. Pressure-based bite transducers using pneumatic scaffolds and fluid-filled deformable chambers offer enhanced adaptability by more evenly distributing occlusal forces across curved dental arches^58,208^. Unlike orthodontic force sensors, which are confined to research prototypes, bite force sensors have been deployed in population-level studies evaluating correlations between craniofacial morphology and bite strength^209,210^. However, limited dynamic range and response lag hinder its use in real-time diagnostics or time-resolved studies of mastication dynamics. To overcome these limitations, next-generation systems have incorporated ultra-thin piezoelectric film sensors (~0.1 mm), offering both high spatial and temporal resolution^211,212^.

Beyond functional bite, bruxism concentrates on the same engineering requirements: nocturnal events are brief, clustered, and can include peaks that approach or exceed daytime voluntary effort^213^. Clinically, recent cohort work links bruxism with higher maximum bite force and greater rates of tooth or restoration fractures, and calls for bite force methods to guide prevention and treatment, strengthening the case for instrumented force monitoring^214^. Based on these engineering requirements for bruxism, the bite force sensor designed for the application of bruxism emphasizes these performance indicators: dynamic range and impact robustness, form factor and comfort, and responsibility. To satisfy these constraints in practice, mouthguard and splint carriers have become the dominant form^215^. Pressure-sensitive polymer stacks with on-board thresholding and time stamping support multi-night episode logging at low average power, whereas ultrathin electret and dielectric-elastomer films add self-biased or self-powered sensing in submillimeter laminates for prolonged wear^216^. And flexible PVDF films offer high sampling rates and the option of small arrays to map where loads concentrate^217^.

## Conclusions and future directions

Recent advances in biosensor technology have demonstrated substantial potential for detecting a broad spectrum of oral biomarkers, including ions (e.g., sodium, potassium, fluoride), small molecules (e.g., glucose, lactate), pH, microbial species, and proteins. These analytes are instrumental in deepening our understanding of oral biology and enhancing the early diagnosis, monitoring, and management of oral diseases. While many biosensors have shown robust analytical performance and long-term stability in vitro, their integration into clinical settings remains a critical hurdle.

Emerging platforms developed for other biofluids, such as sweat, interstitial fluid, and gastrointestinal secretions, offer valuable insights and design strategies that may be adapted for intraoral applications. However, the oral cavity presents unique challenges, including a highly dynamic biochemical environment influenced by diet and hygiene routines, constant mechanical motion, and broad temporal and physiological variability. These conditions demand bio-interfaces that are not only highly sensitive and selective but also mechanically durable, biocompatible, and minimally obtrusive.

Most current efforts in oral biosensing have focused on developing proof-of-concept devices, with limited demonstrations of clinical utility or integration into standard care workflows. A more integrated research and development effort is needed to address commercialization and regulatory barriers that hinder translation. On the commercialization side, sensor designs that not only optimize analytical performance, but also consider scalable manufacturing processes and optimize user acceptance, comfort, and long-term adherence are needed. On the regulatory side, pathways for intraoral biosensors in constant contact with the oral mucosal tissues or saliva are still emerging. Demonstrating long-term safety, reliability, and data integrity will be critical for approval and adoption. A key next step will be the systematic generation of high-quality, clinically relevant data that can validate the impact of continuous, real-time oral biomarker monitoring on diagnosis, treatment, and patient outcomes. Ultimately, bridging the gap between technological innovation and clinical translation will be essential to fully realize the promise of biosensing in advancing precision oral healthcare.

**Table 9:** Non-cytokine protein biosensors

Analyte	Recognition element	Readout signal	Matrix	Dynamic range	Ref
MMP-2					
	Antibody	Electrical	Buffer	6.9 × 10^−6^ ~ 0.7 nM	^125^
	Antibody	Electrical	Buffer	1.4 × 10^−6^ ~ 0.139 nM	^126^
MMP-7					
	Polypeptide	Electrical	Buffer	0.4 ~ 42 nM	^127^
MMP-8					
	Antibody	Acoustic	Saliva	0.2 ~ 21 nM	^128^
	Antibody	Optical	Saliva	0.05 ~ 6.3 nM	^129^
MMP-9					
	Antibody	Electrical	Buffer	1.1 × 10^−7^ ~ 10.9 nM	^124^
CRP					
	Antibody	Electrical	Saliva	0.05 ~ 1 nM	^120^
ALP					
	Cell	Electrical	Saliva	1 ~ 150 μM	^122^

## Data Availability

No datasets were generated or analysed during the current study.
